# Economic and Accessible Portable Homemade Magnetic Hyperthermia System: Influence of the Shape, Characteristics and Type of Nanoparticles in Its Effectiveness

**DOI:** 10.3390/ma17102279

**Published:** 2024-05-11

**Authors:** Teresa Castelo-Grande, Paulo A. Augusto, Lobinho Gomes, Ana Rita Castro Lopes, João Pedro Araújo, Domingos Barbosa

**Affiliations:** 1 LEPABE—Laboratory for Process Engineering, Environment, Biotechnology and Energy, Faculty of Engineering, University of Porto, Rua Dr. Roberto Frias, 4200-465 Porto, Portugal; anaritacastrolopes@gmail.com (A.R.C.L.); dbarbosa@fe.up.pt (D.B.); 2 Instituto de Biología Molecular y Celular del Cáncer, CSIC/Universidad de Salamanca (GIR Citómica), 37001 Salamanca, Spain; pauloaugusto@usal.es; 3 CEADIR—Centro de Estudios Ambientales y Dinamización Rural, Universidad de Salamanca, 37008 Salamanca, Spain; 4 Faculdade de Ciências Naturais, Engenharias e Tecnologias, Universidade Lusófona do Porto, 4000-098 Porto, Portugal; 5 IFIMUP—Institute of Physics for Advanced Materials, Nanotechnology and Photonics, Physics Department, Faculty of Sciences, University of Porto, 4169-007 Porto, Portugal

**Keywords:** magnetic hyperthermia, magnetic nanoparticles, SAR measurements

## Abstract

Currently, one of the main causes of death in the world is cancer; therefore, it is urgent to obtain a precocious diagnosis, as well as boost research and development of new potential treatments, which should be more efficient and much less invasive for the patient. Magnetic hyperthermia (MH) is an emerging cancer therapy using nanoparticles, which has proved to be effective when combined with chemotherapy, radiotherapy and/or surgery, or even by itself, depending on the type and location of the tumor’s cells. This article presents the results obtained by using a previously developed economic homemade hyperthermia device with different types of magnetite nanoparticles, with sizes ranging between 12 ± 5 and 36 ± 11 nm and presenting different shapes (spherical and cubic particles). These magnetic nanoparticles (MNPs) were synthesized by three different methods (co-precipitation, solvothermal and hydrothermal processes), with their final form being naked, or possessing different kinds of covering layers (polyethylene glycol (PEG) or citric acid (CA)). The parameters used to characterize the heating by magnetic hyperthermia, namely the Specific Absorption Rate (SAR) and the intrinsic loss power (ILP), have been obtained by two different methods. Among other results, these experiments allowed for the determination of which synthesized MNPs showed the best performance concerning hyperthermia. From the results, it may be concluded that, as expected, the shape of MNPs is an important factor, as well as the time that the MNPs can remain suspended in solution (which is directly related to the concentration and covering layer of the MNPs). The MNPs that gave the best results in terms of the SAR were the cubic particles covered with PEG, while in terms of total heating the spherical particles covered with citric acid proved to be better.

## 1. Introduction

The World Health Organization (WHO) estimates that in recent decades the total number of people diagnosed with cancer has almost doubled, from about 10 million in 2000 to 19.3 million in 2020. According to the WHO, one in five people in the world will develop cancer during their lifetime, and the recent COVID-19 pandemic has intensified problems concerning a precocious diagnosis and lack of access to treatment [1]. Also, according to the WHO, the number of deaths from cancer has increased from 6.2 million in 2000 to 10 million in 2020. More than one in six deaths worldwide is due to cancer [2].

It is known that an elevated body temperature can damage, or even kill, cancerous cells with minimal injury to normal cells [3]. In the second half of the XIX century, the practice for infectious fever therapy involving hyperthermia was quite common [4]. Hyperthermia is an approach for the treatment of different types of cancer, which involves heating the tissues causing the death of the cancerous cells. Conventionally, these treatments were performed by using ultrasound, microwave or infrared radiation, but these treatments frequently induce heating of the surrounding healthy tissues provoking their damage. Some other methods, like radiofrequency (RF) ablation, have been reported as successful alternative treatments [5]. Nonetheless, some limitations still exist, like the fine control needed to ablate all viable tumor tissue and the definition of an adequate tumor-free margin (an important factor that affects the success of RF thermal ablation) [6]. Hyperthermia has proved to be a selective process for heating tumors when associated with magnetic nanoparticles, which are applied to the tumor site. In 1957, for the first time, Gilchrist reported the use of magnetic nanoparticles (maghemite) to selectively induce temperature elevation and destroy metastatic tumors that were exposed to a 1.2 MHz magnetic field, which was an important discovery that boosted the research on magnetic hyperthermia using magnetic nanoparticles [7]. Nonetheless, the struggle for the development of this technique has been very demanding, and the probability of success by using a specific magnetic material in a given application has proved to be highly dependent on three main factors: (1) the proper synthesis/manufacturing of the magnetic nanosized materials; (2) the correct characterization of the materials, their properties and their functionalities; and, finally, (3) the adequate evaluation of its performance under the specific conditions for the desired application. Consequentially, the exponential growth of research in this area is not surprising, mainly due to its vast possible applications. Different sub-areas have emerged from these studies, such as drug deliver, new synthesis methods [8,9,10], encapsulation [11,12,13] and the functionalization of magnetic nanomaterials [14,15].

Clinical trials by the U.S. Food and Drug Administration (FDA) on the treatment of prostate [16] and pancreatic cancer [17] have already begun. Various hyperthermia techniques are presently under investigation, including local, regional and whole-body hyperthermia [18,19]. But, it is still essential to understand the physical and chemical phenomena that occur during the heating of the human body by magnetic hyperthermia in order for it to be possible to improve both the equipment used for treatment and the techniques used for the production of MNPs, leading to a more secure and efficient treatment by this technique. According to international standards, the maximum values of the field-frequency product (H × f) applied to live organisms should not exceed the upper limit of the Atkinson–Brezovich criterion, that is, H × f ≤ 4.85 × 10^8^ A·m^−1^·s^−1^. On the other hand, regarding the clinical application of magnetic hyperthermia, a safety limit is the Hergt and Dutz criterion that stipulates H × f ≤ 5 × 10^9^ A·m^−1^·s^−1^ [20,21], which is related to the values at which patients start feeling some discomfort.

In the past two decades, the field of research associating nanoscience and nanotechnology has arisen great interest in the scientific community, due to its large range of applications. The exceptional scientific interest in nanomaterials is due to their differentiated properties when compared to those of a bulk material and the discrete atomic or molecular species from which they derive. This interest extends to a diversity of areas, such as [22,23] catalysis, biology, biomedicine and optico-electronic devices, among others. Regarding the area of biomedicine, research on possible applications of nanomaterials as carriers for controlled drug delivery, the separation of proteins and cells, the detection of bacteria and multimodal image processing probes stands out. The differentiated properties of nanoparticles (NPs), such as optical properties in semiconductor and metal-oxide NPs, and the phenomenon of superparamagnetism in ferromagnetic or ferrimagnetic nanomaterials are consequences of a reduced size and arise as a function of some factors, such as high surface/volume ratio, high surface energy, spatial confinement and quantum size effects. Thus, these properties are a function of size (i.e., they are size-tunable). In the past decade, research works devoted to the association of techniques (dual therapies) are increasingly being reported [24]. Some works concerning economic analysis of the production of particles specially designed for biomedical applications have also started to appear [25].

The composition of the nanoparticles used in hyperthermia has been increasing in variety and many NPs are magnetite-based (being bare-naked or functionalized and covered with a specific layer of another material, e.g., citric acid, alginate, etc.); moreover, lately there has been a focus on the use of ferrites (e.g., Mn-Ferrites [26,27].

The improvement of magnetic hyperthermia therapies relies on four major factors: (i) the engineering of alternating magnetic field generating devices, (ii) the engineering of nanostructured magnetic materials, (iii) the development of nanocarriers and other delivery strategies and (iv) the establishment of treatment protocols. Success in each of these steps depends deeply on the synergies of four areas of knowledge: the physical process of heat generation, the heat transfer process, temperature monitoring and the biological effects of temperature at this level.

This work focusses on the first three areas, and for that, different types of magnetic nanoparticles were produced to determine the overall efficiency of a new developed magnetic hyperthermia device, and the best synthesis route for the production of MNPs that give the best performance when applied to magnetic hyperthermia.

### 1.1. Magnetism and Heat Generation

The capability of magnetic materials exposed to an alternating magnetic field of inducing a local temperature increase results from magnetic energy dissipation. In addition, this heating caused by energy loss under a high-frequency alternating current (AC) magnetic field is majorly related to three types of physical mechanisms: currents induced in the material, energy loss through hysteresis and magnetic relaxation of single-domain superparamagnetic nanoparticles (*Néel and Brown*, relaxation phenomena). Recently, another source of heat (the magnetocaloric effect) is being studied for several applications, but for magnetite particles and for the range of frequencies applied in magnetic hyperthermia, its contribution to the total heat is negligible [28,29,30]. The mechanisms of currents induced in the material, and of the energy loss through hysteresis, have a small contribution to total heating when considering single-domain nanoparticles or nanoparticles with a superparamagnetic character. The third mechanism (magnetic relaxation of single-domain superparamagnetic nanoparticles—*Néel and Brown*, relaxation phenomena) and its main influence for local warming is related to the phenomenon of magnetic relaxation of superparamagnetic nanoparticles that are immersed in a fluid medium. There are two mechanisms related to this magnetic relaxation: the Brown rotational mode and the Néel mode. In *Néel* mode, heating comes from changing the orientation of the moment of magnetization of the superparamagnetic nanoparticle from an easy axis of magnetization into the direction of the external magnetic field. This type of mechanism can be compared to the hysteresis energy loss of *multi-domain nanoparticles*. In *Brownian* mode, local heating is due to friction between superparamagnetic nanoparticles and the fluid medium during vibration in the same direction as that of the external magnetic field [31]. A final mention should be carried out to the influence of temperature on the magnetocrystalline anisotropy of the particles when the hyperthermia process is in progress: on the one hand, its effect through hysteresis losses is usually reduced to negligible levels because they present a very low ferromagnetism character or only a superparamagnetic character (due to the reduced size of the particles); on the other hand, in what concerns the changes on the anisotropy constant (*K_eff_*), it is important to notice that in the same type of particle, also bearing in mind the reduced range of temperatures handled in magnetic hyperthermia (between 20 and 46 °C, at the most), the value of the anisotropy coefficient does not suffer considerable changes [32,33].

The amount of heat generated per unit volume is given by the frequency multiplied by the area of the hysteresis loop (adapted from [34]):

(1) $$P_{MF}=-\mu_{0}\times f\oint HdM$$

where $P_{MF}$ is the power absorption density of the magnetic fluid, $\mu_{0}$ = 4π × 10^−7^ N/A^2^ is called the permeability of free space in a magnetic field of frequency f, *M* is the magnetization, *H* the applied magnetic field and ∮HdM the dynamic hysteresis loop determination.

Equation (1) already includes Brown and Néel mechanisms and ignores other possible mechanisms for magnetically induced heating, such as eddy current heating and ferromagnetic resonance, but these are generally irrelevant in the present context. Indeed, the applied AC field frequencies are usually too low for the generation of any substantial eddy currents and the particles used for magnetic hyperthermia are very small. Also, for the frequencies usually used in magnetic hyperthermia, ferromagnetic resonance effects are irrelevant. Comparing the specific loss power of different types of magnetic iron oxide particles (multi-domain or single-domain superparamagnetic nanoparticles), hysteresis losses have been shown to vary according to the specific type of magnetite particles, and in the case of field amplitudes below 10 kA.m^−1^, the difference can reach orders of magnitude due to differences in particle microstructure, shape and size.

### 1.2. SAR Calculation [35,36]

Several methods have been proposed for the calculation of the Specific Absorption Rate (SAR) [35,36,37]. The theoretical background for SAR calculation is an important feature that will be detailed together with two of the most appropriate practical equations and methods used for its calculation.

The background for SAR calculation is based on basic concepts of Magnetism and Thermodynamics. In fact, the application of the General Principle of Conservation of Energy in an adiabatic system allows for the determination of the change that occurs in the internal energy of the suspension, which is associated with the irreversible work caused by the interaction of the system with the applied magnetic field. For an isolated system, the total energy is considered constant regardless of the changes that occur. The equation that translates this phenomenon is as follows:

(2) $$∆U_{susp}=W+Q_{susp}$$

where *W* is the irreversible work carried out on the suspension, ∆*U_susp_* the variation in the internal energy of the suspension and *Q_susp_* the heat that is lost by the suspension. The total work is the result of two types of work: the mechanical work due to changes in volume (*W_mec_*), i.e., boundary work, and the work carried out on the suspension caused by the interaction between the alternating magnetic field and the magnetic nanoparticles (*W_mag_*). By considering the process as adiabatic (*Q_susp_* = 0) and isochoric (*W_mec_* = 0), Equation (2) turns into

(3) $$∆U_{susp}=W_{mag}=C_{susp}\times ∆T$$

where ∆*T* is the temperature change observed in the suspension and *C_susp_* the heat capacity of the suspension, determined generically by

(4) $$C_{susp}=\sum_{j}^{n}m_{j}\times C_{j}$$

where *C_j_* and *m_j_* are the specific heat and mass of the *j*th constituent of the suspension, respectively, and *n* the total number of constituents in the suspension (magnetic nanoparticles and fluid). Therefore, the specific loss power (SLP) may be computed as the generated power (Energy/∆*t*) per unit mass of magnetic material (magnetic nanoparticles), where ∆*t* is a time interval. If the system operates isochoric and adiabatically, the *SLP* may be determined by the following:

(5) $$SLP=\frac{1}{m_{MNP}}\times \frac{W_{mag}}{∆t}=\frac{\frac{∆U_{susp}}{∆t}}{m_{MNP}}≅\frac{1}{m_{MNP}}\times C_{susp}\times {\left(\frac{dT}{dt}\right)}_{t\to 0}$$

This equation is one of the most accepted and used ones by several authors [35,36] for SLP calculation. However, it has the limitation of being valid only in quasi-adiabatic processes (i.e., temperature of the system considered to vary linearly with time and the system being thermally isolated), which is not true because the suspension is not completely isolated in real applications.

There is still debate about the difference between the SAR and the SLP, with the most accepted view being the consideration that the SAR corresponds to the heat actually absorbed by the biological media per gram of nanoparticles, and the SLP the heat produced per gram of nanoparticles, with the SLP thus being the most correct form to address the direct measures using hyperthermia devices. In this article, the position of several authors that consider both as equivalent will be followed, but it is important to understand the possible difference.

Hence, in the case of adiabatic systems, the SAR may be computed by the following:

(6) $$SAR=\frac{d}{dt}\left(\frac{dW_{mag}}{dm}\right)=\frac{d}{dt}\left(\frac{dW_{mag}}{\rho dV}\right)\left[\frac{watts}{g}\right]\;\mathrm{or}\;SAR=C\times {\left(\frac{dT}{dt}\right)}_{t\to 0}\left[\frac{W}{g}\right]$$

or

(7) $${SAR}_{MNP}=C_{MF}\times \frac{∆T}{∆t}\times \frac{1}{\emptyset_{MNP}}\;in\;which\;\frac{1}{\emptyset_{MNP}}=\frac{m_{MF}}{m_{MNP}}$$

or

(8) $$\mathrm{SAR}=\frac{\left(m_{H_{2}O}\times {Cp}_{H_{2}O}+m_{MNP\;}\times {Cp}_{MNP}\right)}{\;m_{MNP\;}}\times \frac{dT}{dt}$$

where *ρ* is the density of the MNPs, *V* is the sample volume, *C* is the heat capacity, *dT/dt|t*→0 is the initial slope of the heating curve, *C_MF_* is the heat capacity of the magnetic fluid, *φ_MNP_* is the percentage of magnetic particles in the sample, *m_MF_* is the mass of the magnetic fluid, *m_MNP_* is the mass of magnetic nanoparticles, $m_{H_{2}O}$ is the mass of the suspending fluid—water, ${Cp}_{H_{2}O}$ is the specific heat of the suspending fluid—water—and *Cp_MNP_* is the specific heat capacity of the magnetic nanoparticles.

#### 1.2.1. Non-Adiabatic Conditions

If a model really wants to correctly translate what happens in a magnetic hyperthermia system, the interactions between the suspension and the surroundings must be taken into consideration. This means that heat interaction between the suspension and its neighborhood should not be neglected, and thus, in Equation (2), Q_susp_ is not negligible, implying a non-adiabatic process. This would be the best approach to describe this process.

Analyzing what happens in the system, the following stages are present and thus should be considered in the model: (a) the suspension is inserted in the sample holder, and the system is in thermal equilibrium with the surrounding environment; (b) when the electrical power is switched on to create a magnetic field, the work resulting from the interaction between the suspension and the alternating magnetic field starts to act on the suspension (W_mag_); (c) the temperature starts to increase in the system, due to the conversion of W_mag_ into internal energy, and an adiabatic system may be assumed to be established, without a considerable approaching error, during a short time interval (i.e., the heat loss Q_susp_ can be neglected during a short period of time), meaning that during this period the assumption of quasi-adiabatic regime is a good approximation and the temperature varies linearly with time; (d) after this initial period, the quasi-adiabatic approximation loses its validity, and thus heat loss cannot be neglected anymore, as an exchange of energy between suspension and surroundings is no longer negligible, and therefore a non-adiabatic regime must be taken into consideration by the model while nonetheless maintaining its isochoric characteristics.

In this case, the SLP may be obtained by

(9) $$SLP={\left.\frac{1}{m_{MNP}}\times C_{susp}\times \frac{dT}{dt}\right|}_{adiabatic}+\frac{1}{m_{MNP}}\times \frac{dQ_{susp}}{dt}$$

(10) $$SLP=\frac{P}{m_{MNP}}$$

where *P* is the total power acting on the suspension taking into account two contributions: the power generated due to the influence of the acting AC magnetic field (which is equal to the total power felt by the suspension in the case of an adiabatic system)—*P_mag_*—and the power lost due to heat transfer (*P_Q_*)—the rate of heat loss from the system to the surroundings. Equation (9) represents the most adequate general representation of the SLP (the actual rate of heat generated per mass of magnetic material in suspension). The difference between Equations (5) and (9) relates to the presence of a second term in the definition of the SLP that considers the non-adiabatic regime. The latter denotes the dependence of the SLP on the rate of heat loss from the system to the surroundings, which may be defined as

(11) $$P_{Q}=\frac{dQ_{susp}}{dt}$$

##### Box–Lucas Method

In an adiabatic system, heat transfer between the sample vessel suspension and the surroundings is not allowed. Naturally, all the heat produced by the movement of MNPs is converted into an increase in sample temperature. In this ideal case, the sample temperature is only dependent on the power generated (P) because the contribution of P_Q_ is equal to zero.

One of the most frequently used approaches for non-adiabatic systems (especially when the power generated by the MNPs is estimated using the heating curve) is considering that the heat loss linearly depends on the difference of temperatures between the surroundings and the sample:

(12) $${\left.C\times \frac{dT(t)}{dt}\right|}_{real}=P_{mag}-L\times ∆T$$

where *L* (W K^−1^) is a proportionality constant relating the heat loss and temperature difference, and Δ*T* is the temperature difference (*T* − *T*_0_), with *T* being the sample temperature at a given time *t* and *T*_0_ the initial sample temperature. From Equation (12), it may be observed that the slope of the heating curve will decrease with time as the temperature of the sample increases until the steady state is reached (i.e., when temperature remains constant, and the energy lost per unit time equals the input of energy). Integrating Equation (12) is as follows:

(13) $$T-T_{0}=\frac{P}{L}\left(1-e^{\left(\frac{t-t_{0}}{\frac{C}{L}}\right)}\right)\Leftrightarrow \;∆T=A\times (1-e^{\left(-B\times ∆t\right)}).\;\mathrm{Usually}\;t_{0}=0\;⇨∆T=A\times (1-e^{\left(-B\times ∆t\right)})$$

In magnetic hyperthermia, this phenomenological equation is commonly referred to as the Box–Lucas equation [36,38,39].

By representing T vs. t, according to Equation (13), the Box–Lucas parameters (A and B) can be determined by curve fitting [36], and the SAR obtained by

(14) $${SAR}_{BLM}=(A\times B\times C_{susp})/(m_{MNP})$$

SAR_BLM_ means SAR obtained by the Box–Lucas method.

The linear loss parameter *L* (W/K) for an individual sample is calculated by the following equation:

(15) $$L=B\times C_{susp}$$

Some authors [36] tried to specify the variables of the Box–Lucas equation by taking into account the rate of heat loss by conduction and convection from the system to the surroundings, obtaining the following formula:

(16) $$T\left(t\right)-T\left(0\right)=∆T\left(t\right)=m_{NMP}\times \frac{SLP}{\in }\times \left(1-e^{-\frac{\in }{C_{susp}}\times t}\right)$$

where ∈ is the effective thermal conductance.

It is important to notice that although the Box–Lucas equation sets a standard for SAR measurement, the linear loss assumption is only valid for low temperature differences, being relatively difficult to justify for real experiments.

Taking into account all possible losses, the relationship between temperature and heat losses is found to be non-linear, especially for higher temperatures. A more comprehensive description of the heating process would be as follows:

(17) $$C\times \frac{dT(t)}{dt}=P-P_{L}(T)$$

This equation contemplates the situations where power loss is assumed as non-linear. In fact, it is based on the fact that heat dissipation is linear in the case of conduction and convection but at the fourth power in the case of radiation. Therefore, usually a fourth-order polynomial approximation is applied to represent function *P_L_*(*T*), which describes the power loss for each temperature. Nonetheless, this equation also has some limitations, namely the fact that in practice the measured *T* or Δ*T* represents a point in the sample and not the temperature experienced by the entire sample (because temperature distribution is non-homogeneous).

The intrinsic loss power parameter (ILP, nH m^2^ kg^−1^) was proposed for the normalization of SAR values measured at different magnetic field amplitudes/frequencies, and it is calculated by the following:

(18) $$ILP=\frac{SAR}{H^{2}\times f}$$

##### Corrected Initial Slope Method

Another approach for the calculation of the SAR is the initial slope method. This method considers that in the initial part of the Box–Lucas temperature–time curve heat losses are negligible, and thus the SAR may be easily calculated by (with β being the slope of the curve)

(19) $$\beta ={\left.\frac{dT}{dt}\right|}_{t\to 0}={\left.\frac{d}{dt}\left(\frac{P}{C}\left(1-e^{-\frac{t}{L/C}}\right)\right)\right|}_{t\to 0}=\frac{P}{C}$$

and thus

(20) $${SAR}_{initial-slope}=\frac{\beta C}{m_{MNP}}$$

This equation may be confirmed as being similar to Equation (6) (obtained for adiabatic systems), as should be expected for the periods when heat losses are considered negligible.

When experiments are carried out, the temperature–time curves are determined for the suspension and for a reference sample composed only of the solvent. To ensure that the heating curve starts at the same temperature as the reference sample—solvent—the measurement of the heating of the suspension of MNPs should start a few seconds later to ensure that steady state has been reached when the actual values start to be registered, and thus the initial solution temperature that is considered is actually equal to the solvent temperature. For actual measurements, a delay in the recording of the SAR/ILP of 5 to 10 s is usually sufficient, although some authors used longer times (such as, for example, 30 s) [40].

A more convenient approach, called the corrected slope method, may be used, in which the previous method is modified to include any linear losses that might be present during the initial stage of the curve. For this correction, when the value of thermal loss, L, of the system is known, the SAR can be calculated using the following:

(21) $${SAR}_{corrected\;slope}=(C\frac{dT}{dt}+L∆T)/(m_{MNP})$$

The SAR and ILP can then be calculated using this corrected slope method.

Another approach to the method has been proposed by Wildeboer and co-workers [39], who stated that for a more accurate calculation of the parameters the heating curve should be divided into N intervals (intervals spanning between 30 and 60 s) which will allow for the computation of both the SAR/ILP parameters for each interval and then the computation of the overall average value as well as the linear loss parameter L. The following formula describes the mathematical procedure for the corrected slope method:

(22) $$SAR=\frac{1}{N}\sum_{i}^{N}\frac{c({\frac{dT}{dt})}_{i\;}+L{\left(∆T\right)}_{i}}{m_{MNP}}$$

in which *C* is the heat capacity of the sample (J K^−1^), and $({\frac{dT}{dt})}_{i\;}$ is the slope obtained by a linear fit of the data in the chosen interval.

## 2. Experimental Section

### 2.1. Equipment

**Figure 1:**
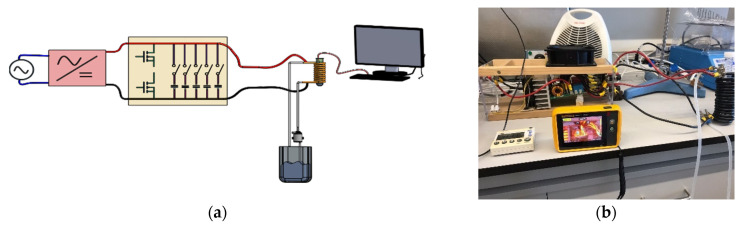
(**a**) General scheme of the homemade system and (**b**) the constructed homemade system.

Generally, an induction heating system consists of a resonant circuit and an AC power supply (providing radiofrequency current). The electrical current is generated by either a DC-AC inverter or an amplifier-connected function generator, with the resonant circuit being in series or in parallel. Soft switching is the most efficient and typical technique used for DC-AC inversion. Several topologies using the soft-switching technique have been proposed, e.g., full-bridge and half-bridge. These topologies usually employ the metal-oxide semi-conductor field-effect transistor (MOSFET) device for switching. The circuit of the system proposed in the present work is an RLC (Resistor–Inductor–Capacitor) system described in [41] and represented in Figure 1. In this setup, the resonant frequency of the tank circuit (regardless of its components) is directly followed; therefore, it can be used with different induction coil formats which allows for obtaining different frequencies. In the present study, the application of a versatile, low-cost, homemade setup for magnetic hyperthermia studies is reported, using magnetic nanoparticles with different shape and composition, which were synthesized by different methods.

### 2.2. Materials and Methods

Metal-oxide nanoparticles, especially magnetic ones, are the most used in biomedical applications due to the possibility of their manipulation by using magnetic fields. The intrinsic properties of these particles that are important for medical applications are their non-toxicity and biocompatibility (*toxicity* refers to the potential harm that may be caused by a material, whereas *biocompatibility* further extends to the detrimental or beneficial effect of the physiological environment on the material performance [42]) and easiness in being injected. Furthermore, nanoparticles also need to be biocompatible and stable in external gravitational and electrostatic fields [43]; in fact, gravitational influence on magnetic particle stability occurs by settling the particles, while electrostatic fields also influence magnetic nanoparticles behavior in solution, usually by promoting their aggregation due to electrostatic attraction. When relevant, both problems are usually solved by the addition of a covering layer that reduces settling and electrostatic attraction (e.g., PEG). The magnetic ground state of nanoparticles can be severely altered, when compared to the conventional assumption that they are single magnetic domains, because it is strongly influenced by the finite size and microstructural details of the core and surface.

The major type of magnetic nanoparticles used in research are iron oxides with a special focus on magnetite (Fe_3_O_4_), which has excellent magnetic properties and is one of the mostly used in the areas of medicine and environment, although metals such as cobalt and nickel are used in other fields of application. The great advantage of these nanoparticles are the chemical modifications that can be made (especially at the surface), making them even more non-toxic, injectable, biocompatible and magnetic in nature, thus turning them into excellent contrast agents. Despite the numerous iron oxides that are known, the term “iron oxides” normally refers to Fe_3_O_4_ (magnetite), α-Fe_2_O_3_ (hematite) and γ-Fe_2_O_3_ (maghemite) [44]. At present, most of the research focuses on magnetic iron oxides such as magnetite (Fe_3_O_4_) or maghemite (γ-Fe_2_O_3_), because iron oxides are the only magnetic nanomaterials approved for use in humans [45,46,47,48]. The general biocompatibility and clinical utility of iron oxides are established by their continuous clinical use, though a detailed understanding of their complex interactions with biological systems continues to evolve [46,49,50,51]. Since the 1980s, various magnetic iron oxide nanoparticle (MIONP) formulations have been used as clinical contrast agents for magnetic resonance imaging (MRI) [52,53]. Indeed, interest has grown exponentially, as is shown by the fact that more than 41 000 research paper with keyword “Fe_3_O_4_” have been published in the past 20 years, according to the Web of Science ([54]).

#### 2.2.1. Materials

This study used the following: iron (III) acetylacetonate (99%) from Sigma Aldrich (St. Louis, MO, USA), oleic acid (90%) from Panreac (Barcelona, Spain), benzyl ether (99%) from Sigma Aldrich (Madrid, Spain), 4-biphenylcarboxylic acid (99%) from Sigma Aldrich (Madrid, Spain), toluene (99.8%) from Panreac (Barcelona, Spain), hexane (99%) from Panreac (Barcelona, Spain), chloroform (99.9%) from Panreac (Barcelona, Spain), nitrogen (99%) from Air Liquide (Paris, France), polyethylene glycol (PEG) from Sigma Aldrich (Madrid, Spain), ferrous sulfate heptahydrate (FeSO_4_·7H_2_O) from Sigma Aldrich (Madrid, Spain), ferric chloride hexahydrate (FeCl_3_·6H_2_O) from Sigma Aldrich (Madrid, Spain), citric acid from Sigma Aldrich (Madrid, Spain) and ammonium hydroxide (25%) from Sigma Aldrich (Madrid, Spain). All reagents were used without further purification.

#### 2.2.2. Synthesis of Nanoparticles

The synthesis of superparamagnetic nanoparticles (SPNPs) is a complex process due to their colloidal nature, and it is challenging to find experimental conditions that produce monodisperse magnetic particles. There are several methods for synthesis, such as thermal decomposition, hydrothermal reactions, microemulsion, and coprecipitation. In terms of simplicity, coprecipitation is the route preferred by researchers, but in terms of controlling the size and morphology of nanoparticles, thermal decomposition seems to be the best method developed so far [11]. Microemulsion can also be used as an alternative to synthesize monodisperse nanoparticles with different morphologies; however, this method requires a large amount of solvent. Hydrothermal synthesis is a relatively unexplored method for the synthesis of MNPs, although it allows for the synthesis of high-quality nanoparticles. Coprecipitation and thermal decomposition for the synthesis of nanoparticles are some of the most studied methods, and although initially the obtained particles differ significantly from method to method, nowadays, both can, within certain limits, be used on a large scale, and the MNPs obtained by these methods have high crystallinity, controlled size and uniform shape (even in the case of coprecipitation, as shown in this work) [11].

##### Synthesis of Cubic-Shaped Magnetite Nanoparticles

In this work, a method for the synthesis of cubic magnetic nanoparticles was developed and applied, which was adapted from Sánchez and coworkers [55]. Typically, 2 mmol of tris(acetylacetonate) iron(III), 4.5 mmol of oleic acid, 52.5 mmol of benzyl ether and 2 mmol of 4-biphenyl reacted in a 3-way flask with magnetic stirring at 200 rpm, in the presence of nitrogen gas, until the temperature raised to 290 °C, and then they were left to react for 30 min. Afterwards, a mixture of toluene and hexane (4:1) was added and the obtained solution was centrifuged at 2500 rpm for 10 min. At the end of the process, the sample was cooled and a mixture of toluene and hexane, in a 4:1 volumetric ratio, was added, and the sample centrifuged at 1700 rpm. Finally, chloroform was used to clean the particles [55].

##### Synthesis of Cubic-Shaped Magnetite Nanoparticles Coated with PEG

The method developed and applied to the synthesis of cubic magnetic nanoparticles coated with PEG was the following: 2 mmol of tris(acetylacetonate)iron(III), 4.5 mmol of oleic acid, 52.5 mmol of benzyl ether, 2 mmol of 4-biphenylcarboxylic acid and 900 mg of PEG were reacted, with magnetic stirring at 220 rpm, in a nitrogen gas environment, and the temperature raised till 290 °C (in 7 min). The temperature was then maintained for 30 min. A mixture of toluene and hexane (4:1) was then added, and the solution obtained centrifuged at 2500 rpm for 10 min. Finally, chloroform was used for cleaning.

##### Synthesis of Magnetite Nanoparticles by Coprecipitation

The following method was developed and applied to produce nanoparticles by coprecipitation: a basic solution of 200 mL (1:1) NH_4_OH/H_2_O and another solution of 100 mL of 6.37 mM of FeSO_4_·7H_2_O in H_2_O was prepared, dispersed by stirring and subjected to ultrasounds. Then, 10 mL of the latter solution was added to the basic solution, at a rate of 1 mL/min, with stirring at 400 rpm. Afterwards, the solution was stirred at 450 rpm for 1 h [56]. Subsequently, the particles were separated with the help of a magnet and washed with water.

##### Synthesis by Coprecipitation of Magnetite Nanoparticles Coated with PEG

In the case of the synthesis of PEG-coated magnetite nanoparticles prepared by coprecipitation, the following procedure was developed and applied: a solution of 100 mL of 0.01 M ferric chloride hexahydrate was added to 100 mL of 0.05 M ferrous sulfate heptahydrate, mixed at 400 rpm, and heated to 50 °C for 5 min. Then, 100 mL of ammonium hydroxide was added, at 10 mL/minute, with stirring at 400 rpm, and the obtained solution heated to 80 °C. Afterwards, the obtained solution was dried at 50 °C for 24 h. Approximately, 450 mg of nanoparticles were obtained, to which a solution of 450 mL of water and 20 mL of Tween solution was added (this solution was sonicated for 20 min at 40 °C). The previous solution, already with the nanoparticles, was subjected to ultrasounds for 10 min at 40 °C, and then 900 mg of PEG were added and sonicated for 10 min at 47 °C. Subsequently, a basic solution of ammonium hydroxide was added, and the pH increased from 7.2 to 10.4. Finally, the particles were washed until a pH of 7 was reached and then dried at 50 °C for 24 h.

##### Synthesis by Coprecipitation of Magnetite Nanoparticles Coated with Citric Acid

The process developed and applied consisted of the following: A solution of 3 mM of FeCl_2_·7H_2_O and 14 mM of FeCl_3_·6H_2_O was prepared in 180 mL of distilled water, under a nitrogen environment. After complete dissolution of the mixture at room temperature, 50 mL of NaOH was added dropwise to the reacting mixture, which was stirred at 650 rpm and maintained for 10 min at 65 °C, under continuous vigorous stirring. To prevent agglomeration of Fe_3_O_4_, 150 mL of 0.05 M citric acid was added to the reaction mixture, which was then stirred for 10 min (at 65 °C). The nanoparticles were then separated with a magnet and washed with water. Finally, the nanoparticles were redispersed in distilled water after sonication for 5 min.

## 3. Results and Discussion

### 3.1. Nanoparticle Characterization

It is important to notice that in this article only the full characterization of the particles that gave better values of the SAR is presented, as they will be the relevant ones for the aimed application. Nonetheless, it was decided to present the process of synthesis used (Section 2) and the results obtained (Section 3.2) for the remaining particles in order to disclose this information to researchers interested in further pursuing their studies.

#### 3.1.1. Phase Identification and Particle Morphology

**Figure 2:**
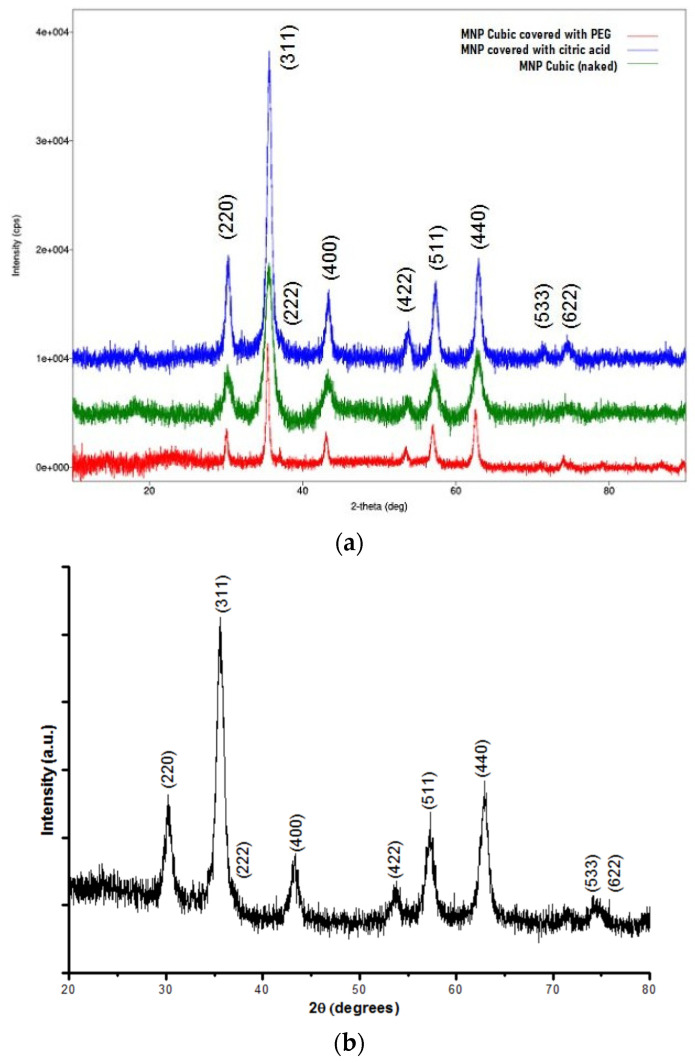
(**a**) X-ray diagrams obtained for the three relevant types of nanoparticles synthesized (blue top line—spherical-shaped particles covered with citric acid; green middle line—naked cubic particles; red bottom line—cubic particles covered with PEG). (**b**) Typical X-ray diffractogram of magnetite/Fe_3_O_4_ [57].

An X-ray diffraction analysis was performed to determine the composition and the chemical species contained in the sample and to characterize the crystals (crystallinity level and, indirectly, size of crystals), knowing that high diffraction peaks indicate a high number of crystal compounds in the sample, while the width of the diffraction peak indicates the size of the crystalline compounds in the sample. The nanoparticles were analyzed by powder X-ray diffraction (XRD) using a Rigaku SmartLab X-ray diffractometer (Rigaku, Tokyo, Japan) with Cu K α radiation (1.5418 Å). For each nanomaterial, the data were collected from 2θ = 3° to 90°. Figure 2 shows the XRD patterns of the obtained magnetite (Fe_3_O_4_) for three selected types of nanoparticles: the cubic-shaped particles—covered and naked—and the spherical-shaped nanoparticles covered with citric acid. The identified peaks are consistent with the results presented in the literature [47]. The XRD diffraction peaks are characteristic of magnetite Fe_3_O_4_ (JCPDS file, No. 00-011-0614)—it may be observed that there is one physical phase due to the presence of magnetite typically in (311); moreover, 2θ of 24.2°, 33.2°, 35.6°, 40.9° and 49.4° corresponding to Fe_3_O_4_ is shown in the XRD patterns in Figure 2. An estimation of the magnetite nanoparticles’ size has been performed using the Scherrer formula. According to the Scherrer equation, the average particle size, D = λ/(β*cos θ), can be estimated from the CuK_α_(λ) X-ray wavelength, Bragg angle (θ) and the total peak width at half height (β) in radian, with λ being the X-ray wavelength (0.15406 nm) and K the shape parameter, which is 0.89 for magnetite. The size ranges of the synthesized magnetite crystals, using the Scherrer equation, were 7.70 ± 0.05, 15.00 ± 0.08 and 20.40 ± 0.06 nm, which are consistent with the SEM image data. Taking the highest intensity peak, namely the (311) plane, at 2θ = 35.70 ± 0.01, and the half maximum intensity width of the peak after accounting for instrument broadening, the calculated crystallite sizes for the relevant types of crystals are presented in Table 1.

#### 3.1.2. SEM

**Figure 3:**
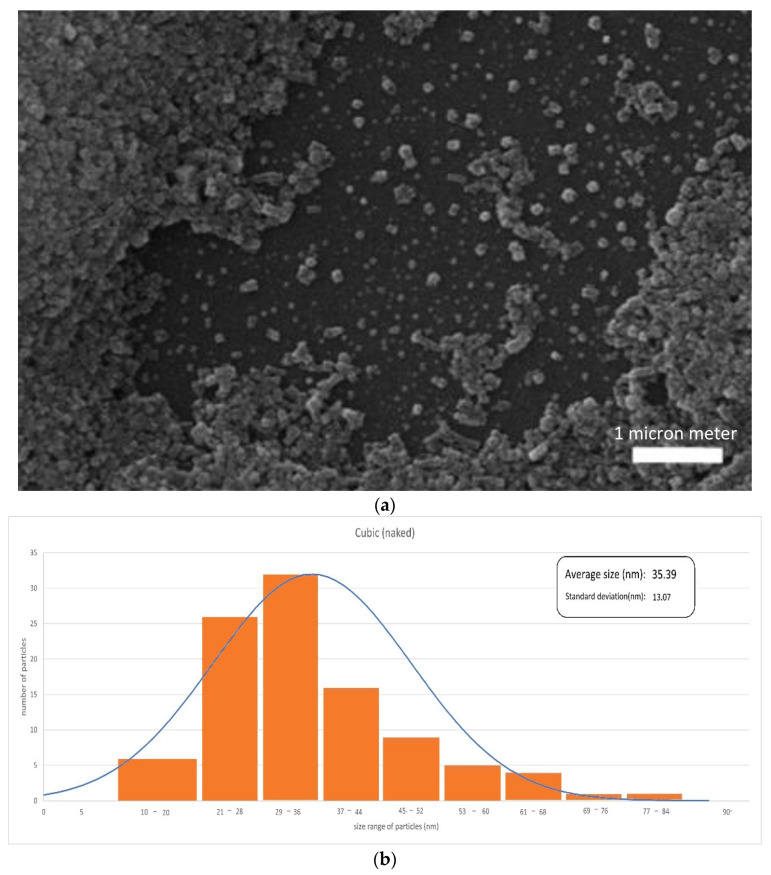
SEM micrograph (**a**) and histogram (**b**) obtained for naked cubic-shaped particles. (Data based on [55]).

**Figure 4:**
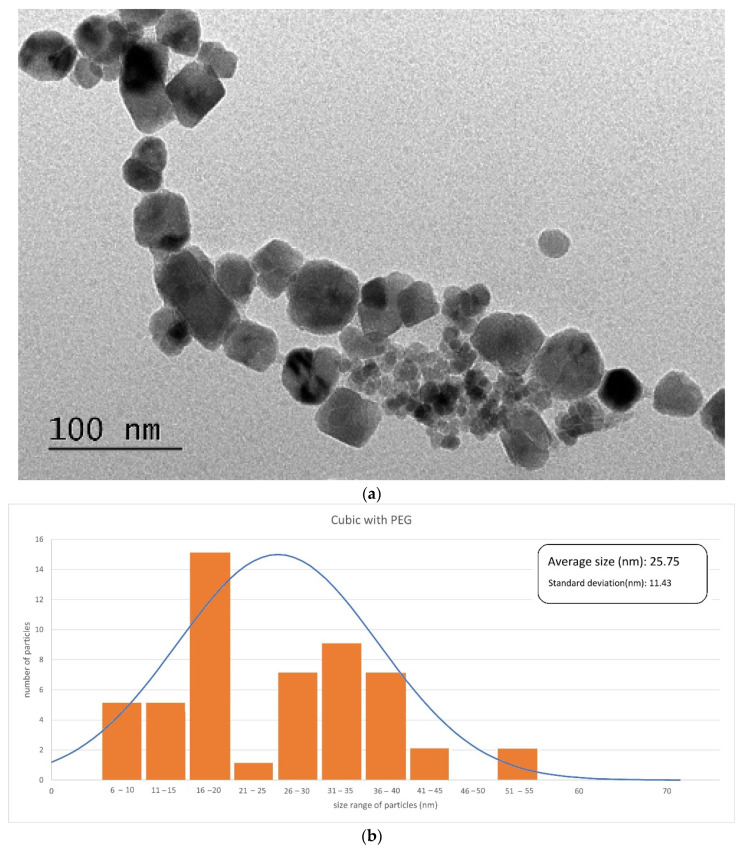
TEM micrograph (**a**) and histogram (**b**) obtained for cubic-shaped particles covered with PEG.

**Figure 5:**
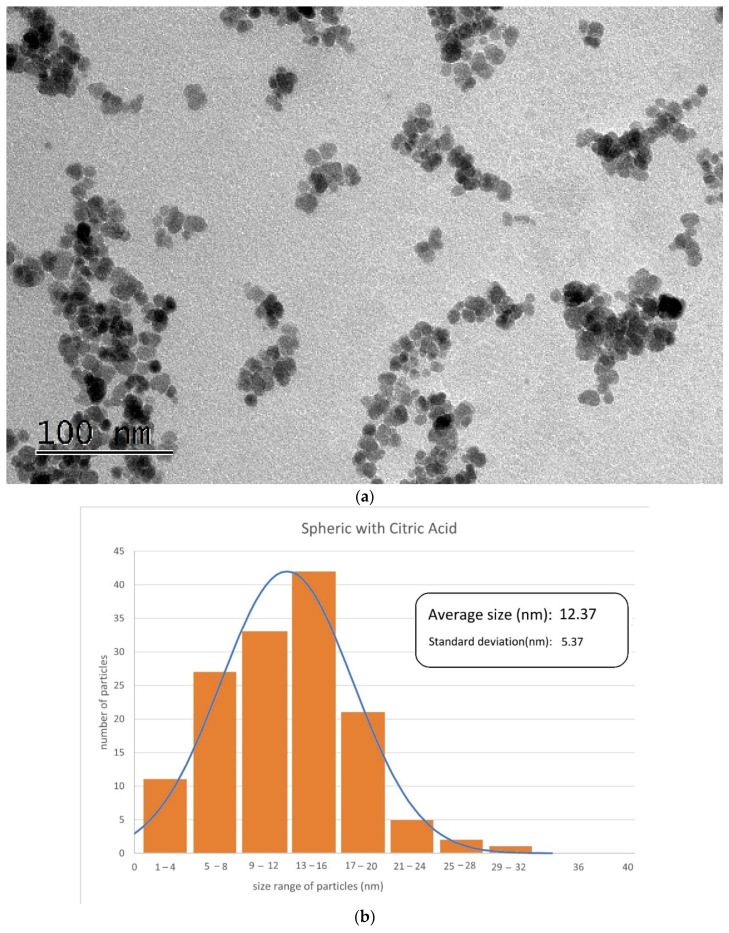
TEM micrograph (**a**) and histogram (**b**) obtained for spherical-shaped particles covered with citric acid.

Figure 3, Figure 4 and Figure 5 illustrate the SEM or TEM micrographs for the best behaved magnetite nanoparticles. These images show that the samples consist of particles with a nearly spherical or cubic shape, depending on the synthesis process. They are approximately 35 nm in size for cubic-shaped naked particles [55], 26 nm for cubic-shaped particles covered with PEG and 12 nm for spherical particles covered with citric acid. The results are in good agreement with those of the previously published literature (e.g., the addition of citrate ions in the co-precipitation solution [58,59] induces low sizes for the diameter of the citrate-coated nanoparticles). The magnetite particles show some kind of agglomeration, even when they are covered with citric acid, which may be justified in this latter case by the formation of a monolayer coverage of magnetite which is reached, for example, in the presence of PEG by coordinating FeOH sites via one or two of their carboxylate functionalities through a water bridge with an outer sphere chemisorption complexation). Nonetheless, the image of coated nanoparticles demonstrates, as expected, that the covered particles are smaller in size as compared to that of the uncoated nanoparticles, because PEG and citric acid can control the particle size by minimizing their agglomeration due to their non-magnetic nature.

#### 3.1.3. Magnetic Characterization

Table 2 presents a sum-up of the main magnetic characteristics of the selected particles, as well as their average size.

**Figure 6:**
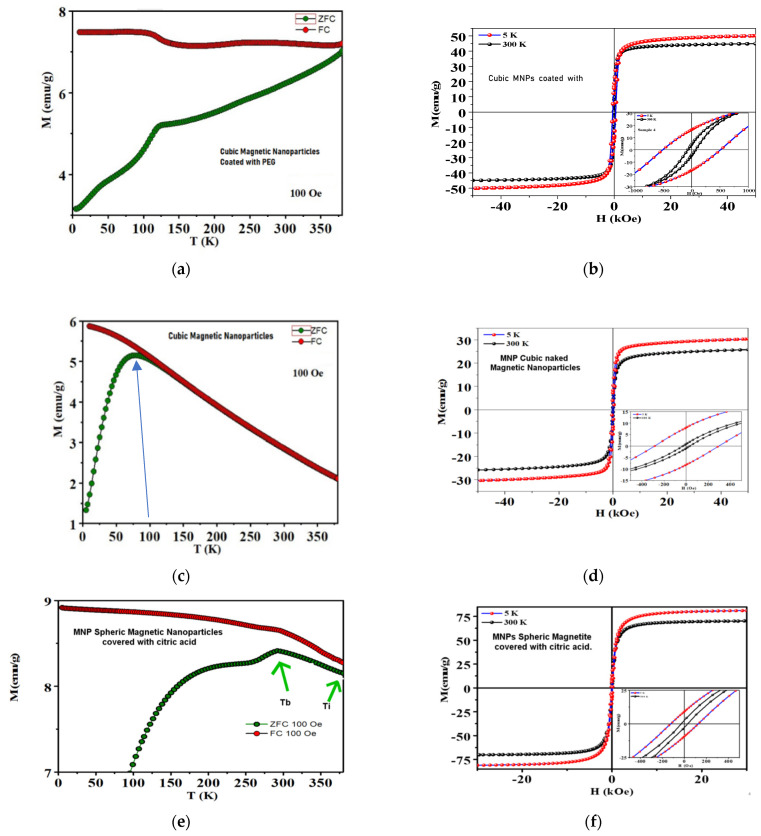
(**a**,**c**,**e**): example of ZFC and FC curves at 100 Oe from 5 to 300 K displaying the blocking temperature (T_b_), irreversibility temperature (T_i_). (**b**,**d**,**f**): magnetization curves at 5 K and 300 K from 40 kOe to −40 kOe for the cubic MNPs and cubic MNPs coated with PEG.

The observed zero-field-cooled (ZFC) and field-cooled (FC) magnetization curves in a field of 100 Oe from 5 K to 300 K are shown in Figure 6. From the observation of the zero-field-cooling and field-cooling curves, it can be concluded that none of the particles are precisely superparamagnetic, which requires that the $\frac{M_{R}}{M_{s}}\;$ ratio ≅ 0 [60], with *M_R_* being the Remanence and *M_s_* the saturation magnetization. The $\frac{M_{R}}{M_{s}}\;$ ratio (which signifies the extent of ferro/ferrimagnetism) for the cubic MNPs coated with PEG and the MNPs coated with CA was 0.32 and 0.12, respectively, indicating that they are ferromagnetic. For the cubic naked particles, the results from the ZFC and FC curves (especially at 100 K—their blocking temperature) indicate that they are apparently superparamagnetic. It is evident that the specific magnetization rises gradually up to a maximum and then decreases with increasing temperature. The temperature at which specific magnetization reaches a maximum, T_max_, is known as the blocking temperature of the system. The blocking temperature (T_B_) of the MNPs covered with CA (325 K) is much higher than that of naked cubic (100 K) and cubic ones covered with PEG (125 K), indicating that MNPs covered with CA have a larger core size. Larger particle size means higher magnetic anisotropy energy, and hence higher thermal energy is required for the superparamagnetic transition. The irreversibility temperatures (T_irr_) obtained for spherical particles are much higher than for cubic samples (the irreversibility temperature is an essential characteristic of the superparamagnetic system [61], above which the ZFC and FC curves superimpose in both samples; generally, in a system containing mono-size and non-interacting superparamagnetic nanoparticles, T_irr_ coincides with T_max_ and the ZFC magnetization curve shows a sharp peak; from the broad maximum of the ZFC curve and from the separation between T_irr_ and T_max_, the particle distribution and interaction between the nanoparticles may be inferred [62,63]).

The MNPs covered with CA show the highest M_s_ value among these samples at 5 K. A high value of M_s_ can make MNPs convert more electromagnetic energy into heat energy under an applied AMF, which can partially explain the higher SAR value of these particles.

A result that was not expected was the detected cubic ZFC/FC curve related with hysteresis losses as a superparamagnetic behavior would be expected without any hysteresis and with higher saturation magnetization. It seems that something is not allowing for the movement of spins, and a possible explanation can be that MNPs agglomerate and form dipoles that prevent the movement of spins, thus provoking a low hysteresis. This is in line with the higher values observed for M_s_ of cubic particles with PEG published in the literature [64] as compared with those observed for naked cubic particles, and this is also corroborated by SEM analysis where it can be seen that agglomerations in little groups are present (enough, on the other hand, to justify the observed polydispersity of size distributions). In the case of MNPs covered with CA, the value of M_s_ is in agreement with what was expected for this type of particles [65]. Relative to cubic MNPs covered with PEG, it is possible to see that there is a dispersion in sizes and the crystal size is bigger than that for the naked cubic MNPs, which may imply a mono-domain presenting hysteresis. The hysteresis curve for MNPs covered with CA was not surprising as from the ZFC magnetization curve it was already concluded that they showed a larger core size, and they presented some polydisperse sizes. Furthermore, the closeness of T_irr_ and T_max_ for the naked cubic sample is associated with some narrow size distributions of the particles, as observed by the SEM analysis, leading to the existence of less magnetic interactions between the particles as compared to those of the CA sample. The magnetic interactions between the particles contribute to an additional energy into effective anisotropy and require higher thermal energy for superparamagnetic transition. Therefore, the blocking temperature shifts towards the higher temperature values in both cubic with PEG and covered CA samples. Several researchers have reported higher blocking temperatures in systems with interacting superparamagnetic nanoparticles [66].

The spherical particles were covered with citric acid in order to eliminate agglomeration of the nanoparticles and thus produce a superior magnetic fluid hyperthermia agent. The surface of the nanoparticles was modified with carboxylic groups by coating with citric acid. The behavior observed during the experiments indicated that the citric acid coating greatly improved the stability of nanoparticles in a water medium for a longer time, which was reflected in the SAR values.

In Table 3, the mean magnetic susceptibility values of the selected particles are presented.

#### 3.1.4. FTIR Analysis

**Figure 7:**
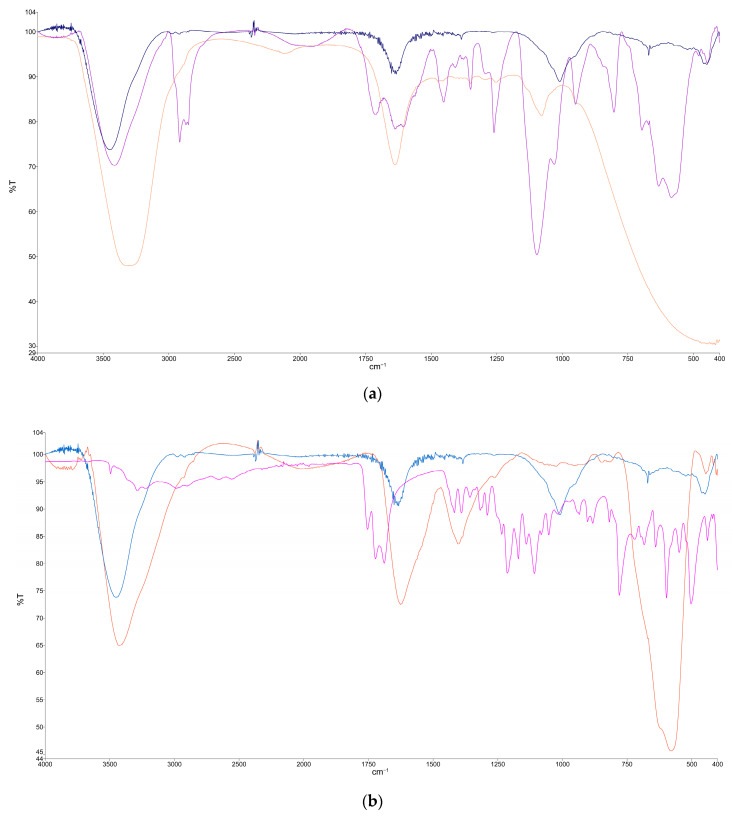
FTIR analysis: (**a**) red line—PEG powder, purple line—cubic MNPs covered with PEG, dark blue line—naked magnetite MNPs; (**b**) red line—spheric MNPs covered with citric acid, dark blue line—naked magnetite MNPs, pink line—citric acid powder.

To analyze the effectiveness of the coating of the MNPs, an FTIR analysis was carried out and compared for these types of nanoparticles. The results are shown in Figure 7.

Although a deeper analysis of the FTIR results will be detailed in a following article that will focus only on the MNP preparation, a preliminary analysis of the results is presented in what follows.

Concerning Figure 7a, the effectiveness of the coating of the MNPs with PEG may be induced by the following observations: there is an absorption band at 1260 cm^−1^, assigned to C–H twisting in PEG; there is also the C–O–C ether stretching absorption band at 1097 cm^−1^ and the bands at ~2800–3000 cm^−1^, corresponding to –C–H symmetric and asymmetric stretching vibrations; the band around 2918 cm^−1^ is also observed, corresponding to –CH_2_ stretching vibrations; and the C–O–C, –CH_2_ and –CH peaks confirm the tethering of PEG onto the Fe_3_O_4_ NP surface [67]. The peaks located at 576 and 646 cm^−1^ are related to the vibration of the Fe-O band in the surface structure of MNPs. The FTIR spectra also confirms the existence of an Fe-O stretching vibration in the range frequency of 658 cm^−1^–506 cm^−1^. Therefore, the peak at 576 cm^−1^ was specified as the characteristic peak of magnetite [68].

With regard to Figure 7b, the effectiveness of the coating of the MNPs with citric acid (CA) may be induced by the following observations: a large and intense band appears at 3429 cm^−1^ that could be assigned to the structural OH groups; the 1700 cm^−1^ peak assignable to the C=O vibration (symmetric stretching) from the COOH group of citric acid (CA) shifts to an intense band at about 1600 cm^−1^ for the ferromagnetic phase coated with citric acid (FF-CA), revealing the binding of a CA radical to the magnetite surface; and the high-intensity bands between 400 and 600 cm^−1^ can be associated with the stretching and torsional vibration modes of magnetite [69,70]. The broad-band spectrum at 3384 cm^−1^ can be referred to as the OH band groups and the traces of molecular water. The 1722 cm^−1^ spectrum peak of CA is due to the symmetric C=O stretching from the COOH group. This peak displays shifts to a lower wavelength at approximately 1615 cm^−1^ for the carboxylic group (R-OOH) of Fe_3_O_4_@CA. The peak at 1615 cm^−1^ determines the binding of the CA radical on the surface of Fe_3_O_4_ NPs through the chemisorption of carboxylate citrate ions [71,72]. The peak at 1384 cm^−1^ can be ascribed to the asymmetric stretching of C–O from the carboxylic group. The intense peak observed in the IR range at approximately 578 cm^−1^ in Fe_3_O_4_@CA could be assigned to the Fe–O stretching vibrational mode of Fe_3_O_4_ [73]. Hence, CA binds to the Fe_3_O_4_ surface through carboxylate.

### 3.2. Hyperthermia Experiments

In this work the behavior of the synthesized MNPs was studied using the previously designed and built homemade hyperthermia apparatus under moderate amplitudes, H_0_ = 8 ± 2 kA and frequencies between 100 and 400 kHz of an AC magnetic field, values that are suitable for clinical applications [74,75]. Even though the SAR values obtained for the five synthesized MNPs were adequate, only the three particles that gave better results (i.e., higher SAR values) were fully characterized (Section 3.1).

**Figure 8:**
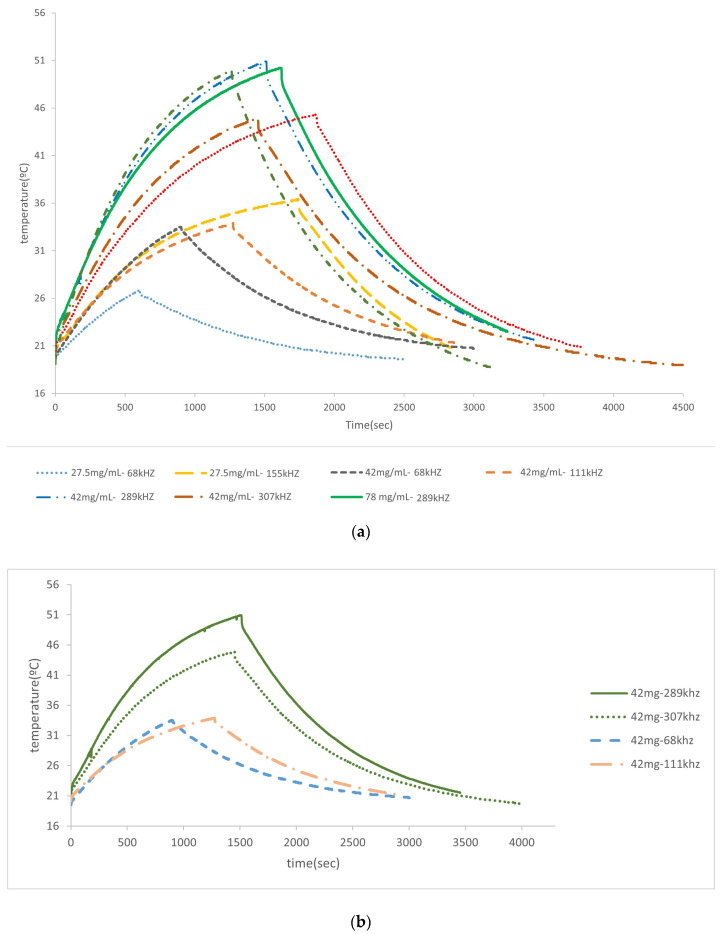
Hyperthermia experiments (spherical particles covered with citric acid). (**a**) with different concentration of particles and magnetic field frequencies; (**b**) for a concentration of 42 mg/mL at different magnetic field frequencies.

The influence of the frequency and concentration of MNPs in the heating curve was studied for the nanoparticles covered with citric acid (see Figure 8). Table 4 shows that when frequencies are increased from 68 kHz to 155 kHz, an increase in the heating and in the value of the SAR (2.55–3.47 W/g) is observed. For higher concentrations (42 mg/mL) at low frequencies, the behavior is superior, reaching a high value of heating at 289 kHz; a further increase in frequencies (307 kHz) makes the generated heating and the corresponding curve decrease, which may indicate that the most effective frequency is probably within the range of 100–290 kHz, which is in agreement with the literature [76,77]. A higher concentration—78 mg/mL—was studied for the same frequency range of 289–307 kHz, and the same result was obtained. Then, the frequency decreased, the concentration changed to 137.6 mg/mL and the obtained curve was very similar to the one obtained for 42–78 mg/mL at 289 kHz. From all these results, it is clear that the frequency that produces the best results (in terms of values of the SAR) is in the range of 155–290 kHz, and even when the number of MNPs is increased, the heating does not seem to improve.

It is possible to confirm this result by looking at Figure 8b, where the curves used for the same concentration (42 mg/mL) are collected, with only the frequency varying.

**Figure 9:**
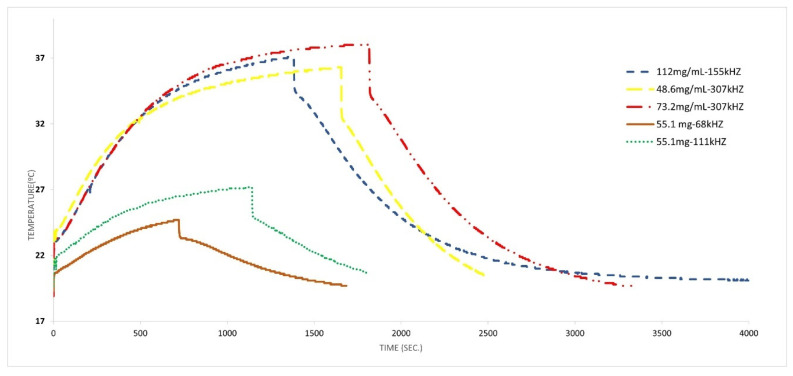
Effect of concentration and frequency in the hyperthermia experiments with cubic particles.

In Figure 9, the case of cubic MNPs is shown. It may be seen that the effect of an increase in frequencies is more important than the effect of an increase in concentration. On the other hand, it may be observed that at the frequency of 307 kHz, if the concentration is almost doubled, the heat does not change significantly; thus, this may be the best frequency for this type of particle, which is in agreement with the literature [78]. Except for the case of very high concentrations, low frequencies are associated with lower heating, and an increase in the frequency implies an increase in the generated heating (for low frequencies).

**Figure 10:**
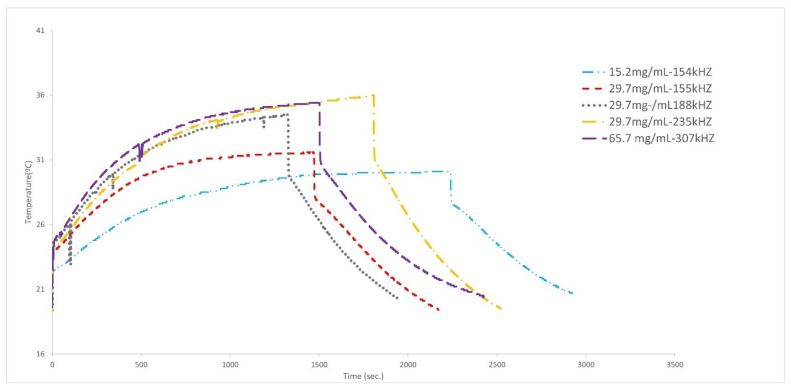
Effect of concentration and frequency in the hyperthermia experiments with cubic nanoparticles covered with PEG.

For the cubic MNPs covered with PEG (Figure 10), it is observed that at low concentrations (15.2 and 29.7 mg/mL) and low frequencies the change in size does not significantly affect the generated heat. When the concentration is doubled, the heating decreases even when frequency increases (65.7 mg/mL at 307 kHz). Thus, it is apparent that for this type of particles the best frequency is situated in the 235 kHz region, and moderate concentrations are better than high concentrations.

**Figure 11:**
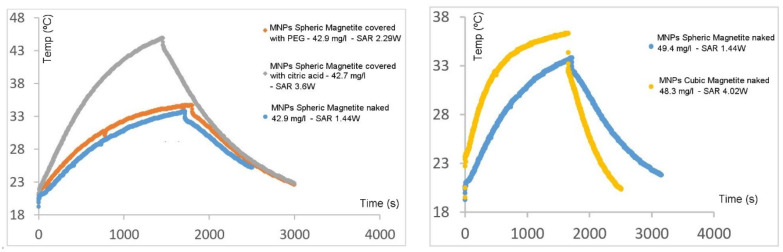
SAR results for the various MNPs at different frequencies, magnetic field and concentration. Cop-c-PEG means spherical MNPs covered with PEG, Cop-c-ac-citric means spherical MNPs covered with citric acid, Cop-s-ver means naked spherical MNPs and Cub-s-ver means cubic naked MNPs.

In Figure 11 and in Table 4, the temperature versus time and SAR values obtained for several of the particles synthesized in this work are shown. As may be seen, the three selected types of particles (cubic–naked, cubic–PEG and spherical–citric acid) show large superior values of the SAR when compared to those of the other two types of particles produced in this work (spherical–PEG and spherical–naked), justifying why the characterization and the core studies are mainly shown for the first three types of synthesized particles.

As far as the results depicted in Table 4 are concerned, it may be concluded that cubic MNPs coated with PEG are the particles that consistently present higher SAR values, even at different frequencies and concentrations, which is in agreement with results published in the literature [78]. In cubic particles covered with PEG, the dipole–dipole interactions are probably weaker and the magnetic heat dissipation is larger. They also present an optimum value for the frequency (between 155 and 189 kHz) for which particles show the best SAR value. In the cases where heating is almost similar, regardless of the absolute values for the concentration of the MNPs, SAR values are always higher for low-concentration samples, as the SAR value is inversely proportional to the mass of the particles. Spherical MNPs coated with citric acid also present very interesting values, although they are much more dependent on the frequency value and on concentration, the best values of the SAR being obtained at frequencies in the range of 155–289 kHz and at low to moderate concentrations. Cubic naked MNPs are more modest concerning SAR values, which are only considerable at high frequencies and low concentrations.

## 4. Conclusions

In the past two decades, nanoparticle-mediated magnetic hyperthermia has witnessed some significant advances, including the development of more robust heat transfer models which nowadays are well established and well supported by experimental results. The synthesis of different magnetic nanoparticles with controlled size and shape has been carried out successfully in this work, and several types of particles produced were able to achieve high SAR values (cubic naked MNPs, cubic MNPs covered with PEG and spherical MNPs covered with citric acid), specially within certain ranges of AC magnetic field frequencies (usually between 100 and 289 kHz) and with low to moderate concentrations. Typical maximum SAR values between 4 and 5 W/g were obtained for the three selected types of particles. A high challenge in this field lies in understanding the role of collective behaviors such as aggregation and agglomeration. In this work, it was possible to better suspend the MNPs producing a higher heating. It was also shown that particle shape influences its performance. Future progress should focus on production and control improvement of even more monodispersed nanoparticles, and on the measurement of their properties, such as anisotropy. There is a clear need for theoretical and experimental advances on this front. In particular, experiments are necessary that may demonstrate the influence of shape in well-dispersed nanoparticle suspensions across a broad range of concentrations and a range of particle sizes and types. A possible next step could be the synthesis of cubic MNPs with narrow size that are able to become very well suspended in a fluid media, so that it may be possible to understand some behaviors and conclude what are the current obstacles that are still faced by the use of cubic nanoparticles.

## Figures and Tables

**Table 1:** Obtained crystal sizes for the most relevant types of particles.

Type of Particles	Size of Crystals (nm)
Cubic	7.70 ± 0.05
Cubic/PEG	20.40 ± 0.06
Spherical/CA	15.00 ± 0.08

**Table 2:** Magnetic and size characteristics of the selected type of particles (M_s_, M_R_ and H_c_ obtained from 5 K to 300 K).

Type of MNP	Avg. Size (nm)	M_S_ emu/g (5 K/300 K)	M_R_ emu/g (5 K/300 K)	M_R_/M_S_	HC kOe (5 K/300 K)	χ_0_ (emu/g·Oe)
Cubic MNPs	35.39	3.00	8.00	0.27	−337.85	2.37 × 10^−2^
Cubic (100 K)	35.39	25.9	−1.47	−0.01	31.08	4.72 × 10^−2^
Cubic w/PEG	25.75	50.0	16.48	0.32	−667.01	2.75 × 10^−2^
MNPs w/CA	12.37	70.0	8.40	0.12	534.91	1.57 × 10^−2^

**Table 3:** Magnetic susceptibility values of the synthesized particles.

Particles	Susceptibility (m^3^/kg)
MNPs covered with CA	2.34 × 10^−1^
Cubic MNPs	2.34 × 10^−2^
Cubic MNPs covered with PEG	1.40 × 10^−2^

**Table 4:** SAR results for the different MNPs according to different frequency, magnetic field and concentration.

Particles	Frequency (kHz)	Concentration (mg/mL)	T_i_/T_fin_./ΔT (K)	SAR (Increment) (W/g)	SAR (BLM) (W/g)
Cubic MNPs coated with PEG	235	29.7	19.3/35.9/16.6	4.0 ± 0.7	4.4
	189	29.7	19.4/34.5/15.1	5.0 ± 0.9	5.8
	155	29.7	20.4/31.6/11.2	4.0 ± 0.6	5.0
	155	15.2	19.8/30.1/10.3	4.2 ± 0.7	5.4
	307	65.7	19.3/35.4	2.8+ 0.4	3.8
MNPs coated with citric acid	289	42.7	20.2/50.1/29.9	4.1 ± 0.4	4.5
	289	78.2	19.7/50.2/30.5	2.3 ± 0.2	2.5
	307	42.7	20.2/44.9/24.7	3.1 ± 0.3	3.6
	307	78.2	19.5/45.3/25.8	1.6 ± 0.1	1.8
	155	27.5	19.5/36.4	3.5 ± 0.4	4.1
Cubic MNPs (naked)	155	122.0	19.9/37.1	1.1 ± 0.1	1.1
	307	73.2	18.9/38.0	1.6 ± 0.2	1.6
	307	48.6	19.5/36.3	3.3 ± 0.3	4.0

Magnetic field (kA/m) 10 ± 2 KA/m.

## Data Availability

Data are contained within the article.

## References

[1] Corley D.A., Sedki M., Ritzwoller D.P., Greenlee R.T., Neslund-Dudas C., Rendle K.A., Honda S.A., Schottinger J.E., Udaltsova N., Vachani A. (2021). Cancer Screening During the Coronavirus Disease-2019 Pandemic: A Perspective From the National Cancer Institute’s PROSPR Consortium. Gastroenterology.

[2] Sung H., Ferlay J., Siegel R.L., Laversanne M., Soerjomataram I., Jemal A., Bray F. (2021). Global Cancer Statistics 2020: GLOBOCAN Estimates of Incidence and Mortality Worldwide for 36 Cancers in 185 Countries. CA Cancer J. Clin..

[3] van der Zee J., González D., van Rhoon G.C., van Dijk J.D., van Putten W.L., Hart A.A. (2000). Comparison of radiotherapy alone with radiotherapy plus hyperthermia in locally advanced pelvic tumours: A prospective, randomised, multicentre trial. Lancet.

[4] Roussakow S. (2013). The History of Hyperthermia Rise and Decline. Conf. Pap. Med..

[5] Monteiro M.S., Mesquita M.S., Garcia L.M., dos Santos P.R., Viveiros C.C.d.M.d., da Fonseca R.D., Xavier M.A., de Mendonça G.W., Rosa S.S., Silva S.L. (2024). Radiofrequency driving antitumor effect of graphene oxide-based nanocomposites: A Hill model analysis. Nanomedicine.

[6] Gillams A.R. (2005). The use of radiofrequency in cancer. Br. J. Cancer.

[7] Gilchrist R.K., Medal R., Shorey W.D., Hanselman R.C., Parrot J.C., Taylor C.B. (1957). Selective Inductive Heating of Lymph Nodes. Ann. Surg..

[8] Liang Y., Xie J., Yu J., Zheng Z., Liu F., Yang A. (2020). Recent advances of high performance magnetic iron oxide nanoparticles: Controlled synthesis, properties tuning and cancer theranostics. Nano Sel..

[9] Ma Z., Mohapatra J., Wei K., Liu J.P., Sun S. (2021). Magnetic Nanoparticles: Synthesis, Anisotropy, and Applications. Chem. Rev..

[10] Zhu K., Ju Y., Xu J., Yang Z., Gao S., Hou Y. (2018). Magnetic Nanomaterials: Chemical Design, Synthesis, and Potential Applications. Accounts Chem. Res..

[11] Ali A., Shah T., Ullah R., Zhou P., Guo M., Ovais M., Tan Z., Rui Y. (2021). Review on Recent Progress in Magnetic Nanoparticles: Synthesis, Characterization, and Diverse Applications. Front. Chem..

[12] Farhanian D., De Crescenzo G., Tavares J.R. (2018). Large-Scale Encapsulation of Magnetic Iron Oxide Nanoparticles via Syngas Photo-Initiated Chemical Vapor Deposition. Sci. Rep..

[13] Mona L.P., Songca S.P., Ajibade P.A. (2021). Synthesis and encapsulation of iron oxide nanorods for application in magnetic hyperthermia and photothermal therapy. Nanotechnol. Rev..

[14] El-Sherbiny I.M., Arafa K., Fytory M., Ahmadi M., Afkhami A., Madrakian T. (2021). 1—Physical properties, classification, synthesis, and functionalization of magnetic nanomaterials. Magnetic Nanomaterials in Analytical Chemistry.

[15] Wang J., Yao J., Sun N., Deng C. (2017). Facile synthesis of thiol-polyethylene glycol functionalized magnetic titania nanomaterials for highly efficient enrichment of N-linked glycopeptides. J. Chromatogr. A.

[16] Mahmoudi K., Bouras A., Bozec D., Ivkov R., Hadjipanayis C. (2018). Magnetic hyperthermia therapy for the treatment of glioblastoma: A review of the therapy’s history, efficacy and application in humans. Int. J. Hyperth..

[17] Spirou S.V., Basini M., Lascialfari A., Sangregorio C., Innocenti C. (2018). Magnetic Hyperthermia and Radiation Therapy: Radiobiological Principles and Current Practice. Nanomaterials.

[18] Feldman A.L., Libutti S.K., Pingpank J.F., Bartlett D.L., Beresnev T.H., Mavroukakis S.M., Steinberg S.M., Liewehr D.J., Kleiner D.E., Alexander H.R. (2003). Analysis of Factors Associated With Outcome in Patients With Malignant Peritoneal Mesothelioma Undergoing Surgical Debulking and Intraperitoneal Chemotherapy. J. Clin. Oncol..

[19] Tay Z.W., Chandrasekharan P., Chiu-Lam A., Hensley D.W., Dhavalikar R., Zhou X.Y., Yu E.Y., Goodwill P.W., Zheng B., Rinaldi C. (2018). Magnetic Particle Imaging-Guided Heating in Vivo Using Gradient Fields for Arbitrary Localization of Magnetic Hyperthermia Therapy. ACS Nano.

[20] Hergt R., Dutz S. (2007). Magnetic particle hyperthermia—Biophysical limitations of a visionary tumour therapy. J. Magn. Magn. Mater..

[21] Kouzoudis D., Samourgkanidis G., Kolokithas-Ntoukas A., Zoppellaro G., Spiliotopoulos K. (2021). Magnetic Hyperthermia in the 400–1100 kHz Frequency Range Using MIONs of Condensed Colloidal Nanocrystal Clusters. Front. Mater..

[22] Augusto P.A., Castelo-Grande T., Vargas D., Hernández L., Merchán L., Estevez A.M., Gómez J., Compaña J.M., Barbosa D. (2020). Water Decontamination with Magnetic Particles by Adsorption and Chemical Degradation. Influence of the Manufacturing Parameters. Materials.

[23] Augusto P.A., Castelo-Grande T., Estévez A.M., Barbosa D., Costa P.M. (2017). Method to evaluate and prove-the-concept of magnetic separation and/or classification of particles. J. Magn. Magn. Mater..

[24] Pacheco A.R.F., Cardoso B.D., Pires A., Pereira A.M., Araújo J.P., Carvalho V.M., Rodrigues R.O., Coutinho P.J.G., Castelo-Grande T., Augusto P.A. (2023). Development of pH-Sensitive Magnetoliposomes Containing Shape Anisotropic Nanoparticles for Potential Application in Combined Cancer Therapy. Nanomaterials.

[25] Augusto P.A., Castelo-Grande T., Vargas D., Pascual A., Hernández L., Estevez A.M., Barbosa D. (2020). Upscale Design, Process Development, and Economic Analysis of Industrial Plants for Nanomagnetic Particle Production for Environmental and Biomedical Use. Materials.

[26] Bentarhlia N., Elansary M., Belaiche M., Mouhib Y., Lemine O., Zaher H., Oubihi A., Kartah B., Monfalouti H. (2023). Evaluating of novel Mn–Mg–Co ferrite nanoparticles for biomedical applications: From synthesis to biological activities. Ceram. Int..

[27] Gaona-Esquivel A., Hernandez-M D.S., Hernández-Rodríguez Y., Cigarroa-Mayorga O. (2022). The role of Nd as a dopant in Mn_3_O_4_NPs on the heat induction of artificial breast tissue due to the irradiation of microwaves. Mater. Chem. Phys..

[28] Franco V., Blázquez J., Ipus J., Law J., Moreno-Ramírez L., Conde A. (2018). Magnetocaloric effect: From materials research to refrigeration devices. Prog. Mater. Sci..

[29] Korolev V.V., Arefyev I.M., Ramazanova A.G. (2008). The magnetocaloric effect of superfine magnets. J. Therm. Anal. Calorim..

[30] Korolev V.V., Aref’ev I.M., Ramazanova A.G. (2007). The magnetocaloric effect and heat capacity of ferrimagnetic nanosystems: High-dispersity magnetite. Russ. J. Phys. Chem. A.

[31] Laurent S., Dutz S., Häfeli U.O., Mahmoudi M. (2011). Magnetic fluid hyperthermia: Focus on superparamagnetic iron oxide nanoparticles. Adv. Colloid Interface Sci..

[32] Anandhi J.S., Arun T., Joseyphus R.J. (2020). Role of magnetic anisotropy on the heating mechanism of Co-doped Fe_3_O_4_ nanoparticles. Phys. B Condens. Matter.

[33] Nemala H., Thakur J.S., Naik V.M., Vaishnava P.P., Lawes G., Naik R. (2014). Investigation of magnetic properties of Fe3O4 nanoparticles using temperature dependent magnetic hyperthermia in ferrofluids. J. Appl. Phys..

[34] Mazon E.E., Villa-Martínez E., Hernández-Sámano A., Córdova-Fraga T., Ibarra-Sánchez J.J., Calleja H.A., Cruz J.A.L., Barrera A., Estrada J.C., Paz J.A. (2017). A high-resolution frequency variable experimental setup for studying ferrofluids used in magnetic hyperthermia. Rev. Sci. Instrum..

[35] Andreu I., Natividad E. (2013). Accuracy of available methods for quantifying the heat power generation of nanoparticles for magnetic hyperthermia. Int. J. Hyperth..

[36] Papadopoulos C., Efthimiadou E.K., Pissas M., Fuentes D., Boukos N., Psycharis V., Kordas G., Loukopoulos V.C., Kagadis G.C. (2020). Magnetic fluid hyperthermia simulations in evaluation of SAR calculation methods. Phys. Medica.

[37] Lanier O.L., Korotych O.I., Monsalve A.G., Wable D., Savliwala S., Grooms N.W.F., Nacea C., Tuitt O.R., Dobson J. (2019). Evaluation of magnetic nanoparticles for magnetic fluid hyperthermia. Int. J. Hyperth..

[38] Ring H.L., Sharma A., Ivkov R., Bischof J.C. (2020). The impact of data selection and fitting on SAR estimation for magnetic nanoparticle heating. Int. J. Hyperth..

[39] Wildeboer R.R., Southern P., Pankhurst Q.A.A. (2014). On the reliable measurement of specific absorption rates and intrinsic loss parameters in magnetic hyperthermia materials. J. Phys. D Appl. Phys..

[40] Vilas-Boas V., Carvalho F., Espiña B. (2020). Magnetic Hyperthermia for Cancer Treatment: Main Parameters Affecting the Outcome of In Vitro and In Vivo Studies. Molecules.

[41] Castelo-Grande T., Augusto P.A., Gomes L., Calvo E., Barbosa D. (2024). An Economic and accessible portable homemade magnetic hyperthermia system.

[42] Rodrigues S., Dionísio M., López C.R., Grenha A. (2012). Biocompatibility of Chitosan Carriers with Application in Drug Delivery. J. Funct. Biomater..

[43] Dossett J., Totten G. (2014). Nanoparticle heating using induction in hyperthermia. ASM Handbook.

[44] Teja A.S., Koh P.-Y. (2009). Synthesis, Properties, and Applications of Magnetic Iron Oxide Nanoparticles. Prog. Cryst. Growth Charact. Mater..

[45] Healy S., Bakuzis A.F., Goodwill P.W., Attaluri A., Bulte J.W.M., Ivkov R. (2022). Clinical magnetic hyperthermia requires integrated magnetic particle imaging. WIREs Nanomed. Nanobiotechnol..

[46] Soetaert F., Korangath P., Serantes D., Fiering S., Ivkov R. (2020). Cancer therapy with iron oxide nanoparticles: Agents of thermal and immune therapies. Adv. Drug Deliv. Rev..

[47] Huo Y., Yu J., Gao S. (2022). Chapter Nanomagnetism: Principles, Nanostructures, and Biomedical Applications. Synthesis and Biomedical Applications of Magnetic Nanomaterials.

[48] Huo Y., Yu J., Gao S. (2022). Magnetic-mediated hyperthermia for cancer treatment: Research progress and clinical trials. Synthesis and Biomedical Applications of Magnetic Nanomaterials.

[49] Korangath P., Barnett J.D., Sharma A., Henderson E.T., Stewart J., Yu S.-H., Kandala S.K., Yang C.-T., Caserto J.S., Hedayati M. (2020). Nanoparticle interactions with immune cells dominate tumor retention and induce T cell–mediated tumor suppression in models of breast cancer. Sci. Adv..

[50] Murray A.R., Kisin E., Inman A., Young S.-H., Muhammed M., Burks T., Uheida A., Tkach A., Waltz M., Castranova V. (2012). Oxidative Stress and Dermal Toxicity of Iron Oxide Nanoparticles In Vitro. Cell Biochem. Biophys..

[51] Zanganeh S., Hutter G., Spitler R., Lenkov O., Mahmoudi M., Shaw A., Pajarinen J.S., Nejadnik H., Goodman S., Moseley M. (2016). Iron oxide nanoparticles inhibit tumour growth by inducing pro-inflammatory macrophage polarization in tumour tissues. Nat. Nanotechnol..

[52] Bulte J.W.M., Daldrup-Link H.E. (2018). Clinical Tracking of Cell Transfer and Cell Transplantation: Trials and Tribulations. Radiology.

[53] Bulte J.W.M., Kraitchman D.L. (2004). Iron oxide MR contrast agents for molecular and cellular imaging. NMR Biomed..

[54] Nguyen M.D., Tran H.V., Xu S., Lee T.R. (2021). Fe_3_O_4_ Nanoparticles: Structures, Synthesis, Magnetic Properties, Surface Functionalization, and Emerging Applications. Appl. Sci..

[55] Sánchez O.S., Castelo-Grande T., Augusto P.A., Compaña J.M., Barbosa D. (2021). Cubic Nanoparticles for Magnetic Hyperthermia: Process Optimization and Potential Industrial Implementation. Nanomaterials.

[56] Ahmed M., Goldberg S.N. (2011). Basic Science Research in Thermal Ablation. Surg. Oncol. Clin. N. Am..

[57] Mihai C.T., Luca A., Danciu M., Alexa-Stratulat T., Ciobanu R., Tamba B.I., Stanciu G.D., Ciortescu I., Mihai C., Stefanescu G. (2019). In vitro evaluation of novel functionalized nanoparticles for terahertz imaging. Med.-Surg. J..

[58] Mansurov Z., Smagulova G., Kaidar B., Imash A., Lesbayev A. (2022). PAN—Composite Electrospun-Fibers Decorated with Magnetite Nanoparticles. Magnetochemistry.

[59] Dubey S.K., Dey A., Singhvi G., Pandey M.M., Singh V., Kesharwani P. (2022). Emerging trends of nanotechnology in advanced cosmetics. Colloids Surf. B Biointerfaces.

[60] Obaidat I.M., Issa B., Haik Y. (2015). Magnetic Properties of Magnetic Nanoparticles for Efficient Hyperthermia. Nanomaterials.

[61] Saragi T., Permana B., Therigan A., Sinaga H.D., Maulana T., Risdiana R. (2022). Study of Magnetic Properties and Relaxation Time of Nanoparticle Fe_3_O_4_-SiO_2_. Materials.

[62] Duan W.J., Lu S.H., Wu Z.L., Wang Y.S. (2012). Size Effects on Properties of NiO Nanoparticles Grown in Alkalisalts. J. Phys. Chem. C.

[63] Ramana P., Rao K.S., Kumar K.R., Kapusetti G., Choppadandi M., Kiran J. (2021). A study of uncoated and coated nickel-zinc ferrite nanoparticles for magnetic hyperthermia. Mater. Chem. Phys..

[64] Andhare D.D., Patade S.R., Khedkar M.V., Nawpute A.A., Jadhav K.M. (2022). Intensive analysis of uncoated and surface modified Co-Zn nanoferrite as a heat generator in magnetic fluid hyperthermia applications. Appl. Phys. A.

[65] Cheraghipour E., Javadpour S., Mehdizadeh A.R. (2012). Citrate capped superparamagnetic iron oxide nanoparticles used for hyperthermia therapy. J. Biomed. Sci. Eng..

[66] Sadat E., Bud’ko S.L., Ewing R.C., Xu H., Pauletti G.M., Mast D.B., Shi D. (2023). Effect of Dipole Interactions on Blocking Temperature and Relaxation Dynamics of Superparamagnetic Iron-Oxide (Fe_3_O_4_) Nanoparticle Systems. Materials.

[67] Banerjee A., Blasiak B., Pasquier E., Tomanek B., Trudel S. (2017). Synthesis, characterization, and evaluation of PEGylated first-row transition metal ferrite nanoparticles as T 2 contrast agents for high-field MRI. RSC Adv..

[68] Malega F., Indrayana I.P.T., Suharyadi E. (2018). Synthesis and characterization of the microstructure and functional group bond of Fe3o4 nanoparticles from natural iron sand in Tobelo North Halmahera. J. Ilm. Pendidik. Fis. Al-Biruni.

[69] Răcuciu M., Creangă D.E., Airinei A. (2006). Citric-acid-coated magnetite nanoparticles for biological applications. Eur. Phys. J. E.

[70] Dheyab M.A., Aziz A.A., Jameel M.S., Abu Noqta O., Khaniabadi P.M., Mehrdel B. (2020). Simple rapid stabilization method through citric acid modification for magnetite nanoparticles. Sci. Rep..

[71] Arefi M., Miraki M.K., Mostafalu R., Satari M., Heydari A. (2018). Citric acid stabilized on the surface of magnetic nanoparticles as an efficient and recyclable catalyst for transamidation of carboxamides, phthalimide, urea and thiourea with amines under neat conditions. J. Iran. Chem. Soc..

[72] Singh D., Gautam R.K., Kumar R., Shukla B.K., Shankar V., Krishna V. (2014). Citric acid coated magnetic nanoparticles: Synthesis, characterization and application in removal of Cd(II) ions from aqueous solution. J. Water Process. Eng..

[73] Yamaura M., Camilo R., Sampaio L., Macêdo M., Nakamura M., Toma H. (2004). Preparation and characterization of (3-aminopropyl)triethoxysilane-coated magnetite nanoparticles. J. Magn. Magn. Mater..

[74] van Landeghem F.K., Maier-Hauff K., Jordan A., Hoffmann K.-T., Gneveckow U., Scholz R., Thiesen B., Brück W., von Deimling A. (2009). Post-mortem studies in glioblastoma patients treated with thermotherapy using magnetic nanoparticles. Biomaterials.

[75] Pankhurst Q.A., Thanh N.T.K., Jones S.K., Dobson J. (2009). Progress in applications of magnetic nanoparticles in biomedicine. J. Phys. D Appl. Phys..

[76] Wu K., Wang J.-P. (2017). Magnetic hyperthermia performance of magnetite nanoparticle assemblies under different driving fields. AIP Adv..

[77] Shah R.R., Davis T.P., Glover A.L., Nikles D.E., Brazel C.S. (2015). Impact of magnetic field parameters and iron oxide nanoparticle properties on heat generation for use in magnetic hyperthermia. J. Magn. Magn. Mater..

[78] Usov N.A., Nesmeyanov M.S., Gubanova E.M., Epshtein N.B. (2019). Heating ability of magnetic nanoparticles with cubic and combined anisotropy. Beilstein J. Nanotechnol..

